# Protective Vascular and Cardiac Effects of Inducible Nitric Oxide Synthase in Mice with Hyperhomocysteinemia

**DOI:** 10.1371/journal.pone.0107734

**Published:** 2014-09-16

**Authors:** Sanjana Dayal, Ilya O. Blokhin, Rochelle A. Erger, Melissa Jensen, Erland Arning, Jeff W. Stevens, Teodoro Bottiglieri, Frank M. Faraci, Steven R. Lentz

**Affiliations:** 1 Department of Internal Medicine, University of Iowa Carver College of Medicine, Iowa City, Iowa, United States of America; 2 Department of Pharmacology, University of Iowa Carver College of Medicine, Iowa City, Iowa, United States of America; 3 Interdisciplinary Graduate Program in Molecular and Cellular Biology, University of Iowa Carver College of Medicine, Iowa City, Iowa, United States of America; 4 Francois M. Abboud Cardiovascular Center, University of Iowa Carver College of Medicine, Iowa City, Iowa, United States of America; 5 Veterans Affairs Medical Center, Iowa City, Iowa, United States of America; 6 Baylor Institute of Metabolic Disease, Dallas, Texas, United States of America; National Institutes of Health, United States of America

## Abstract

Diet-induced hyperhomocysteinemia produces endothelial and cardiac dysfunction and promotes thrombosis through a mechanism proposed to involve oxidative stress. Inducible nitric oxide synthase (iNOS) is upregulated in hyperhomocysteinemia and can generate superoxide. We therefore tested the hypothesis that iNOS mediates the adverse oxidative, vascular, thrombotic, and cardiac effects of hyperhomocysteinemia. Mice deficient in iNOS (*Nos2−/−*) and their wild-type (*Nos2+/+*) littermates were fed a high methionine/low folate (HM/LF) diet to induce mild hyperhomocysteinemia, with a 2-fold increase in plasma total homocysteine (P<0.001 vs. control diet). Hyperhomocysteinemic *Nos2+/+* mice exhibited endothelial dysfunction in cerebral arterioles, with impaired dilatation to acetylcholine but not nitroprusside, and enhanced susceptibility to carotid artery thrombosis, with shortened times to occlusion following photochemical injury (P<0.05 vs. control diet). *Nos2−/−* mice had decreased rather than increased dilatation responses to acetylcholine (P<0.05 vs. *Nos2+/+* mice). *Nos2−/−* mice fed control diet also exhibited shortened times to thrombotic occlusion (P<0.05 vs. *Nos2+/+* mice), and iNOS deficiency failed to protect from endothelial dysfunction or accelerated thrombosis in mice with hyperhomocysteinemia. Deficiency of iNOS did not alter myocardial infarct size in mice fed the control diet but significantly increased infarct size and cardiac superoxide production in mice fed the HM/LF diet (P<0.05 vs. *Nos2+/+* mice). These findings suggest that endogenous iNOS protects from, rather than exacerbates, endothelial dysfunction, thrombosis, and hyperhomocysteinemia-associated myocardial ischemia-reperfusion injury. In the setting of mild hyperhomocysteinemia, iNOS functions to blunt cardiac oxidative stress rather than functioning as a source of superoxide.

## Introduction

Hyperhomocysteinemia is an established risk factor for cardiovascular disease and stroke [1], [2]. Even relatively mild elevations of plasma total homocysteine (e.g. 10 to 20 µmol/L) are associated with increased cardiovascular risk [1], [2]. Several distinct cardiovascular abnormalities, including endothelial and cardiomyocyte dysfunction, and thrombosis, have been demonstrated in animal models of diet-induced hyperhomocysteinemia [3], [4]. Hyperhomocysteinemia has been proposed to exert its adverse effects in the vasculature in part by generating vascular oxidative stress that diminishes the bioavailability of endothelial-derived nitric oxide (NO) [3]. NO is normally produced in a highly regulated manner by endothelial NO synthase (eNOS; *Nos3*), which is expressed constitutively in vascular endothelium. An inducible isoform of NOS (iNOS; *Nos2*) is expressed in vascular smooth muscle, endothelium, and myocardium in certain pathological conditions such as atherosclerosis, diabetes, and inflammatory disorders [5]–[9]. Experimental overexpression of iNOS in isolated murine blood vessels causes endothelial dysfunction [10]–[12], which may be due to iNOS uncoupling [13], [14]. iNOS uncoupling results in the generation of superoxide rather than NO as the predominant reaction product and iNOS-derived superoxide can limit the bioavailability of endothelial NO [15]. Studies in diabetic mice revealed that deficiency of iNOS or reversal of iNOS uncoupling restores endothelium-dependent relaxation [16], [17] and tolerance to myocardial ischemia/reperfusion (I/R) injury [18], [19]. Collectively, these studies suggest a role for iNOS in the pathophysiology of multiple types of cardiovascular disease.

The potential role of iNOS in the etiology of hyperhomocysteinemia-induced cardiovascular abnormalities has not been directly explored, but several lines of evidence suggest that iNOS expression is upregulated in the setting of elevated homocysteine levels. *In vitro* studies with cultured endothelial and smooth muscle cells treated with homocysteine showed increased expression of iNOS [20], [21]. Similarly, upregulation of iNOS has been detected in the coronary and carotid artery, and kidneys of hyperhomocysteinemic rats [22]–[24]. Additionally, we and others have shown that NOS inhibitors abrogate the increased production of reactive oxygen species (ROS) in carotid and coronary arteries of hyperhomocysteinemic rats or mice [22], [25]. These observations suggest that the upregulation and uncoupling of iNOS, with increased production of superoxide, may be a mechanism that contributes to endothelial dysfunction in hyperhomocysteinemia. Consistent with this notion, impaired endothelium-dependent vasomotor responsiveness in the aorta of hyperhomocysteinemic rats could be reversed by tetrahydrobiopterin, an essential cofactor that promotes NOS coupling [26]. It remains unclear, however, whether iNOS directly and mechanistically contributes to the adverse vascular and cardiac effects of hyperhomocysteinemia.

We utilized a genetic approach to examine the mechanistic role of endogenous iNOS in the vascular phenotype of diet-induced hyperhomocysteinemia in mice. Using iNOS-deficient (*Nos2−/−*) mice, we tested the hypothesis that iNOS contributes to endothelial dysfunction, thrombosis, and myocardial ischemia-reperfusion injury in hyperhomocysteinemia. Contrary to our hypothesis, our data suggest that endogenous iNOS protects against, rather than exacerbates, vascular oxidative stress, endothelial dysfunction, and thrombosis. Our findings also indicate that, during hyperhomocysteinemia, endogenous iNOS prevents myocardial injury by blunting cardiac oxidative stress.

## Materials and Methods

### Mice

This study was carried out in strict accordance with the recommendations in the Guide for the Care and Use of Laboratory Animals of the National Institute of Health. All animal protocols were approved by the University of Iowa Animal Care and Use Committee (approval # 1211243). All surgery was performed under anesthesia (sodium pentobarbital) and all efforts were made to minimize suffering. Male homozygous iNOS deficient (*Nos2−*/−) mice on the C57BL6/J background were obtained from the Jackson Laboratory and were backcrossed to C57BL6/J female mice (Jackson Laboratory) to generate male and female heterozygous iNOS deficient (*Nos2*+/−) breeders. These *Nos2*+/− breeder mice were then intercrossed to generate wild-type (*Nos2*+/+) and *Nos2*−/− littermate mice for study. Genotyping for the wild-type and targeted *Nos2* alleles was performed by polymerase chain reaction (PCR) using the following primer sequences: for the wild-type *Nos2* allele, 5′- TCA ACA TCT CCT GGT GGA AC -3′ and 5′- AGC ACA CAT GCA GAA TGA GTA -3′; for the targeted *Nos2* allele, 5′- AAT ATG CGA AGT GGA CCT CG -3′ and 5′- AGC ACA CAT GCA GAA TGA GTA -3′. Starting from 4 weeks of age, mice were fed either a control diet (LM485, Harlan Teklad) that contains 4.0 g/Kg L-methionine and 6.7 mg/Kg of folic acid, or a high methionine/low folate (HM/LF) diet (TD00205, Harlan Teklad) that contains 8.2 g/Kg L-methionine and 0.2 mg/Kg folic acid [27]. Mice were fed the diets for 5–11 months before study.

### Dilator responses in cerebral arterioles

Dilatation of cerebral arterioles was measured as described previously [25]. Briefly, mice were anesthetized with sodium pentobarbital and ventilated mechanically. A cranial window was made over the left parietal cortex, and a segment of a randomly selected pial arteriole (∼30 µm in diameter) was studied. The diameter of the cerebral arteriole was measured, using a video microscope coupled to an image-shearing device, under control conditions and during superfusion with acetylcholine (1.0 µmol/L and 10.0 µmol/L), nitroprusside (0.1 µmol/L and 1.0 µmol/L), and papaverine (1.0 µmol/L and 10.0 µmol/L).

### Carotid artery thrombosis

Carotid artery thrombosis was induced by photochemical injury as described previously [28]. Mice were anesthetized with sodium pentobarbital (70–90 mg/Kg intraperitoneally) and ventilated mechanically with room air and supplemental oxygen. The right femoral vein was cannulated for the administration of rose bengal. The right common carotid artery was dissected free and carotid artery blood flow was measured with a 0.5 PSB Doppler flow probe (Transonic Systems, Inc) and digital recording system (Gould Ponemah Physiology Platform version 3.33). To induce endothelial injury, the right common carotid artery was transilluminated continuously with a 1.5-mV, 540-nm green laser (Melles Griot) from a distance of 6 cm, and rose bengal (35 mg/Kg) was injected via a femoral vein catheter. Blood flow was monitored continuously for 90 minutes or until stable occlusion occurred, at which time the experiment was terminated. Stable occlusion was defined as the time at which blood flow remained absent for ≥10 minutes.

### Myocardial I/R injury

Regional myocardial I/R injury was performed using a procedure modified from Gandhi et al [29]. Male mice were anesthetized with sodium pentobarbital and supplemented every hour (sodium pentobarbital, 20 mg/Kg IP) as needed to maintain adequate depth of anesthesia. Body temperature was monitored with a rectal probe and maintained at 36.5–37.5°C with a heating pad. A tracheotomy was performed and a volume-controlled Harvard respirator (model 681) was used to mechanically ventilate mice with room air (stroke volume 0.24 mL, 105 strokes per minute). A thoracotomy was performed in the fourth intercostal space, and the left coronary artery was ligated 2 mm below the tip of the left atrium using 8.0 Ethicon mounted on a tapered needle. A short (1 mm) section of PE-10 tubing was placed into the ligature to facilitate later reperfusion. Successful occlusion of the left coronary artery was verified by visual inspection (development of paleness of the anterolateral wall of the left ventricle, color change of left coronary artery from bright-red to violet, and regional akinesis). After occlusion for 30 minutes, reperfusion was induced by cutting the ligature on the top of the PE-10 tubing and was visually confirmed by a return of pink-red color of the anterolateral wall of the left ventricle and brief period of tachycardia.

After 2 hours of reperfusion, the left coronary artery was re-ligated and 0.5 mL of 3% Evans blue dye (TCI America) was slowly injected into the left ventricle by cardiac puncture to delineate the ischemic area at risk (AR). The heart was then rapidly excised, rinsed in ice-cold saline, and serially sectioned perpendicular to its long axis using an acrylic mouse heart slicer matrix (Zivic Instruments). Heart sections (2 mm) were immediately imaged using a digital camera (Nikon Coolpix 5000) for determination of AR and then incubated with 3.0 mL of 1.5% 2,3,5-triphenyltetrazolium chloride (TTC) (GFS Chemicals) in phosphate buffer (0.1 mol/L NaH_2_PO_4_/Na_2_HPO_4_, pH 7.4) for 20 minutes at 37°C to demarcate the viable and nonviable area of necrosis (AN) and imaged. The AR, AN, and total myocardial area (TA) were determined using computer planimetry by a blinded investigator using National Institutes of Health (NIH) Image J (v1.57) software.

### Plasma homocysteine and methionine

Blood was collected by cardiac puncture into EDTA (final concentration 5 mmol/L), and plasma was flash frozen. Plasma total homocysteine, defined as the total concentration of homocysteine after quantitative reductive cleavage of all disulfide bonds [30], was measured by high performance liquid chromatography (HPLC) using ammonium 7-fluorobenzo-2-oxa-1,3-diazole-4-sulphonate (SBDF) fluorescence detection [31]. Plasma methionine was measured by HPLC coupled to fluorescence detection after precolumn derivatization using ο-phthaldialdehyde as described previously [32].

### Detection of ROS

The oxidative fluorescent dye dihydroethidium (DHE) (Molecular Probes) was used to detect ROS in sections of cerebral arterioles as described previously [33]. Frozen transverse sections (10 µm) of cerebral arterioles were incubated with 10 µmol/L DHE for 30 minutes at room temperature in a dark chamber. Fluorescence was detected by laser-scanning confocal microscopy. Data were quantified using Image J software and reported as mean fluorescence relative to *Nos2+*/+ mice fed the control diet.

### Real-time PCR

Levels of mRNA for *Nos2*, neuronal and endothelial nitric oxide synthase (*Nos1* and *Nos3*, respectively), and *18S* were measured by quantitative real-time PCR as described previously [34]. Total RNA was isolated from frozen hearts using Trizol reagent (Invitrogen, Carlsbad, CA) and treated with DNAse I to remove contaminating genomic DNA. RNA (325 ng) was then reverse transcribed using Taqman reverse transcriptase and random hexamer primers. PCR primers and 6-carboxy fluorescein-labeled probes for *18s (Mm03928990_g1)*, *Nos2* (Mm01309892_g1), and *Nos3* (Mm00435204_m1) were purchased from Applied Biosystems. Reverse transcribed cDNA was incubated with TaqMan Universal PCR mix (Applied Biosystems) and PCR primers and probes at 50°C for 2 minutes and then at 95°C for 10 minutes followed by 40 cycles of 95°C for 15 seconds and 60°C for 1 minute using the Applied Biosystems 7700 sequence detection system. For quantitative analysis of mRNA, the comparative threshold cycle (ΔΔC_T_) method [35] was used, with values normalized to *18S* and expressed relative to levels in *Nos2*+/+ mice fed the control diet. Validation experiments were performed to confirm equal amplification efficiency for all primers sets.

### Statistical analysis

Two-way analysis of variance (ANOVA) with the Tukey test for multiple comparisons was used to analyze the effects of *Nos2* genotype and diet on endothelial function, occlusion time, ROS levels, infarct size and troponin levels. The data sets for occlusion time and cardiac ROS did not show normal distributions, therefore the analysis was performed on log transformed values. One way ANOVA was used to compare mRNA levels in cardiac tissue. Statistical significance was defined as a P value <0.05. Values are reported as mean±SE.

## Results

### Deficiency of iNOS does not protect against endothelial dysfunction in hyperhomocysteinemic mice

**Figure 1:**
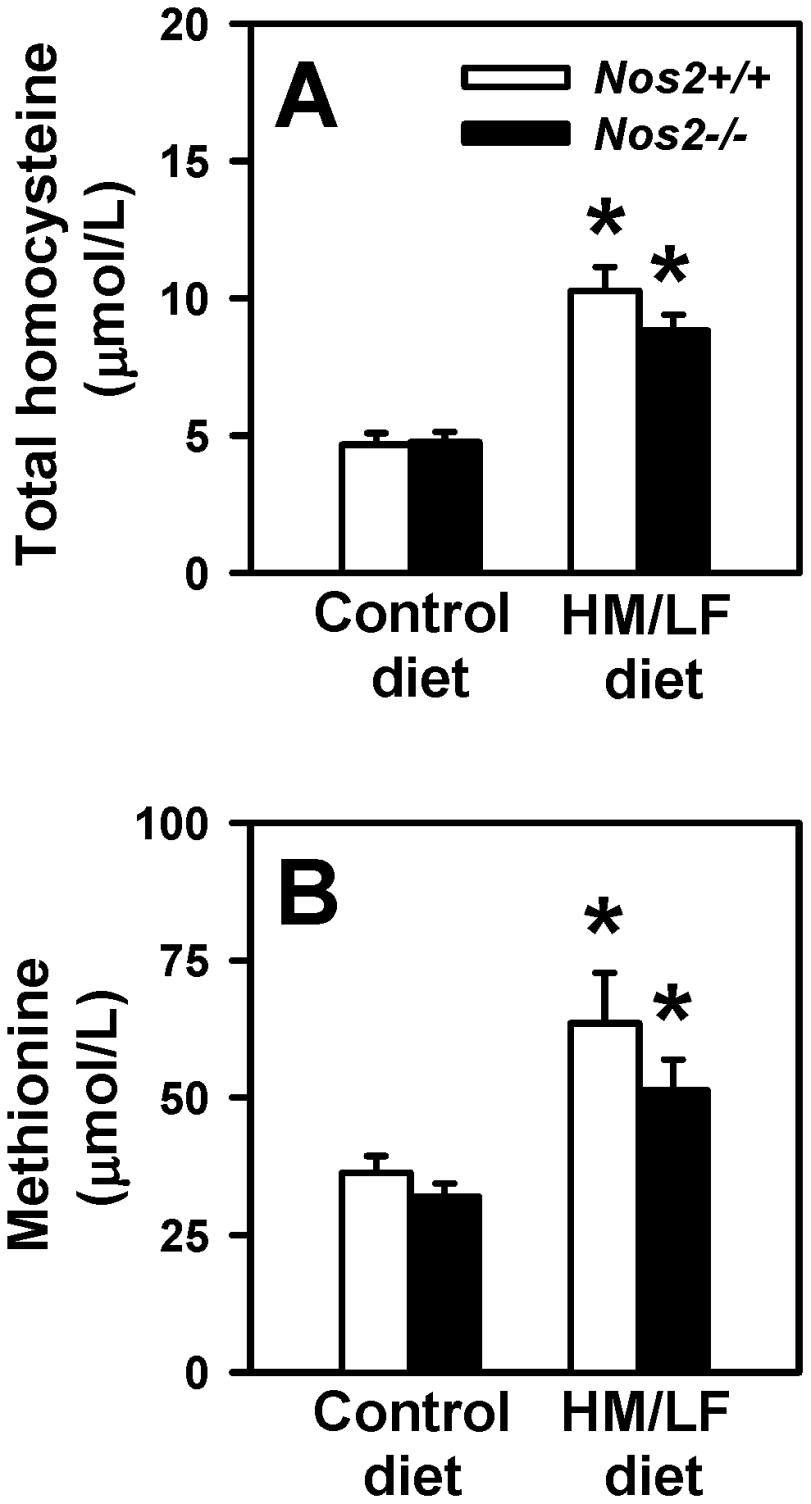
Plasma total homocysteine and methionine levels. Compared with mice fed the control diet, both *Nos2+/+* (open bars) and *Nos2−/−* (filled bars) mice fed the HM/LF diet exhibited elevation in plasma total homocysteine and methionine levels. 15–20 mice were studied in each group. *P<0.01 compared with mice of the same genotype fed the control diet by two-way ANOVA.

To investigate the potential contribution of iNOS to endothelial dysfunction during hyperhomocysteinemia, *Nos2−/−* mice deficient in iNOS were placed on a HM/LF diet. Plasma levels of total homocysteine were elevated approximately 2-fold in both *Nos2+/+* and *Nos2−/−* mice fed the HM/LF diet compared with mice fed the control diet (Figure 1, P<0.001). Plasma levels of methionine also were elevated significantly in mice fed the HM/LF diet compared with mice fed the control diet (Figure 1, P<0.05). Deficiency of iNOS had no impact on plasma levels of homocysteine or methionine (P = 0.5).

**Figure 2:**
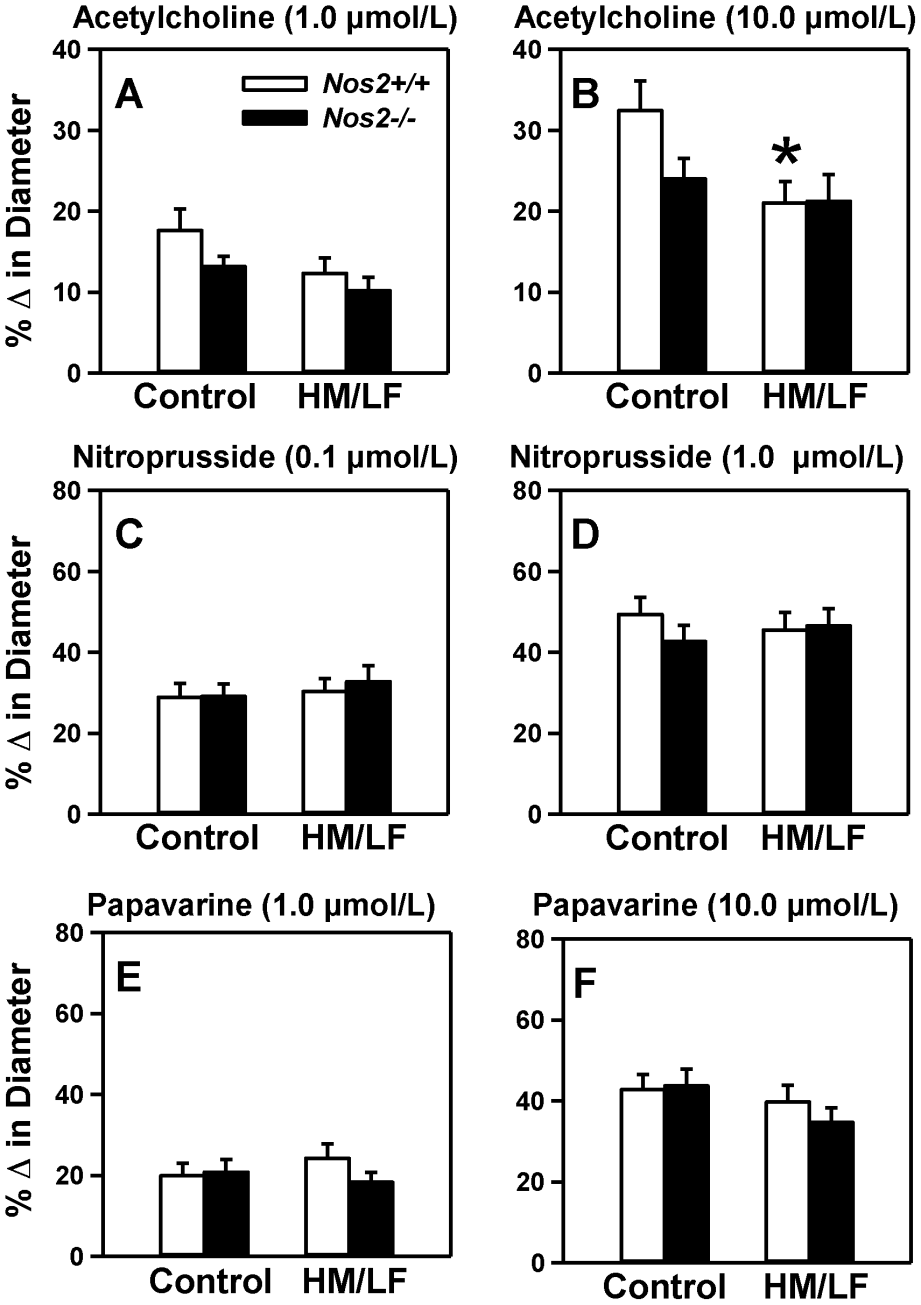
Dilator responses in cerebral arterioles. Dilatation of cerebral arterioles to (A) 1.0 µmol/L acetylcholine, (B) 10.0 µmol/L acetylcholine, (C) 0.1 µmol/L nitroprusside, (D) 1.0 µmol/L nitroprusside, (E) 1.0 µmol/L papaverine and (F) 10.0 µmol/L papaverine were measured in *Nos2+/+* (open bars) or *Nos2−/−* (filed bars) mice. 10–15 mice were studied in each group. *P<0.05 compared with mice of same genotype fed the control diet by two-way ANOVA.

Hyperhomocysteinemia is a risk factor for small vessel disease in the brain [36]. Thus, we next measured responses of small cerebral arterioles to endothelium-dependent and -independent dilators (Figure 2). Dilator responses to 10.0 µmol/L acetylcholine, an endothelium-dependent dilator, were decreased in *Nos2*+/+ mice fed the HM/LF diet compared with *Nos2*+/+ mice fed the control diet (P<0.05; Figure 2B). Deficiency of iNOS failed to protect cerebral arterioles from this effect of hyperhomocysteinemia, as demonstrated by the similar dilator responses to acetylcholine in *Nos2+/+* and *Nos2−/−* mice fed the HM/LF diet. These data suggest that iNOS does not play a causative role in the endothelial vasomotor dysfunction associated with hyperhomocysteinemia. In mice without hyperhomocysteinemia, the data suggest a possible protective effect of endogenous *Nos2*, indicated by a strong trend towards decreased dilator responses to acetylcholine in *Nos2−/−* mice compared with *Nos2+/+* mice fed the control diet, (P = 0.053; Figure 2B). The endothelium-independent vasodilators nitroprusside (Figure 2C and 2D) and papaverine (Figure 2E and 2F) also produced dilatation of cerebral arterioles, and the responses were not influenced by either diet or *Nos2* genotype.

### Absence of iNOS does not reverse the prothrombotic phenotype produced by HM/LF diet

**Figure 3:**
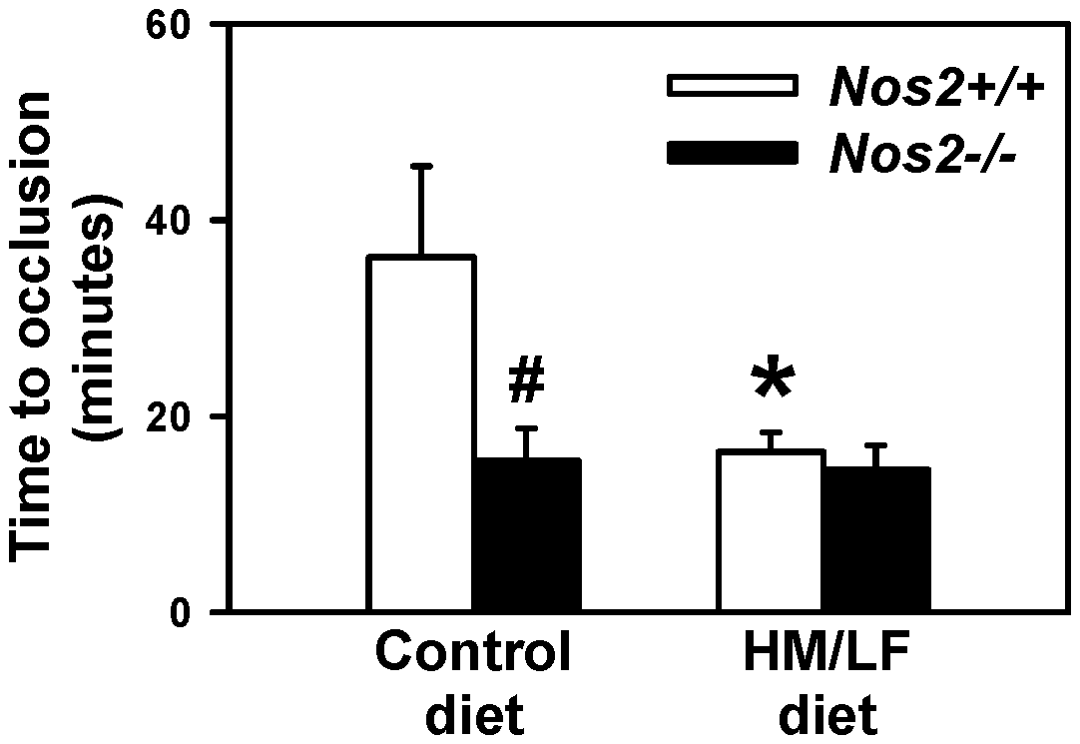
Carotid artery thrombosis. The time to stable occlusion of the carotid artery in *Nos2+*/+ (open bars) or *Nos2−*/− (filled bars) mice fed either the control diet or the HM/LF diet was measured following photochemical injury. 6–8 mice were studied in each group. *P<0.05 compared with mice of the same genotype fed the control diet; ^#^P<0.01 compared with *Nos2*+/+ mice fed the same diet by two-way ANOVA.

To further examine the vascular effects of iNOS deficiency in hyperhomocysteinemic mice, experimental thrombosis of the carotid artery was induced by photochemical injury. *Nos2*+/+ mice fed the HM/LF diet exhibited a significant shortening in the time to stable occlusion compared with *Nos2*+/+ mice fed the control diet (P<0.05, Figure 3). A similar shortening of the time to stable occlusion was observed in *Nos2*−/− mice fed the HM/LF diet, indicating that deficiency of iNOS did not prevent this prothrombotic effect of hyperhomocysteinemia. In mice fed the control diet, the mean time to stable occlusion was significantly shortened in *Nos2−/*− mice compared with *Nos2*+/+ mice (P<0.01), again suggesting a protective effect of endogenous *Nos2*.

### Increased vascular ROS was detected with the HM/LF diet and iNOS deficiency

**Figure 4:**
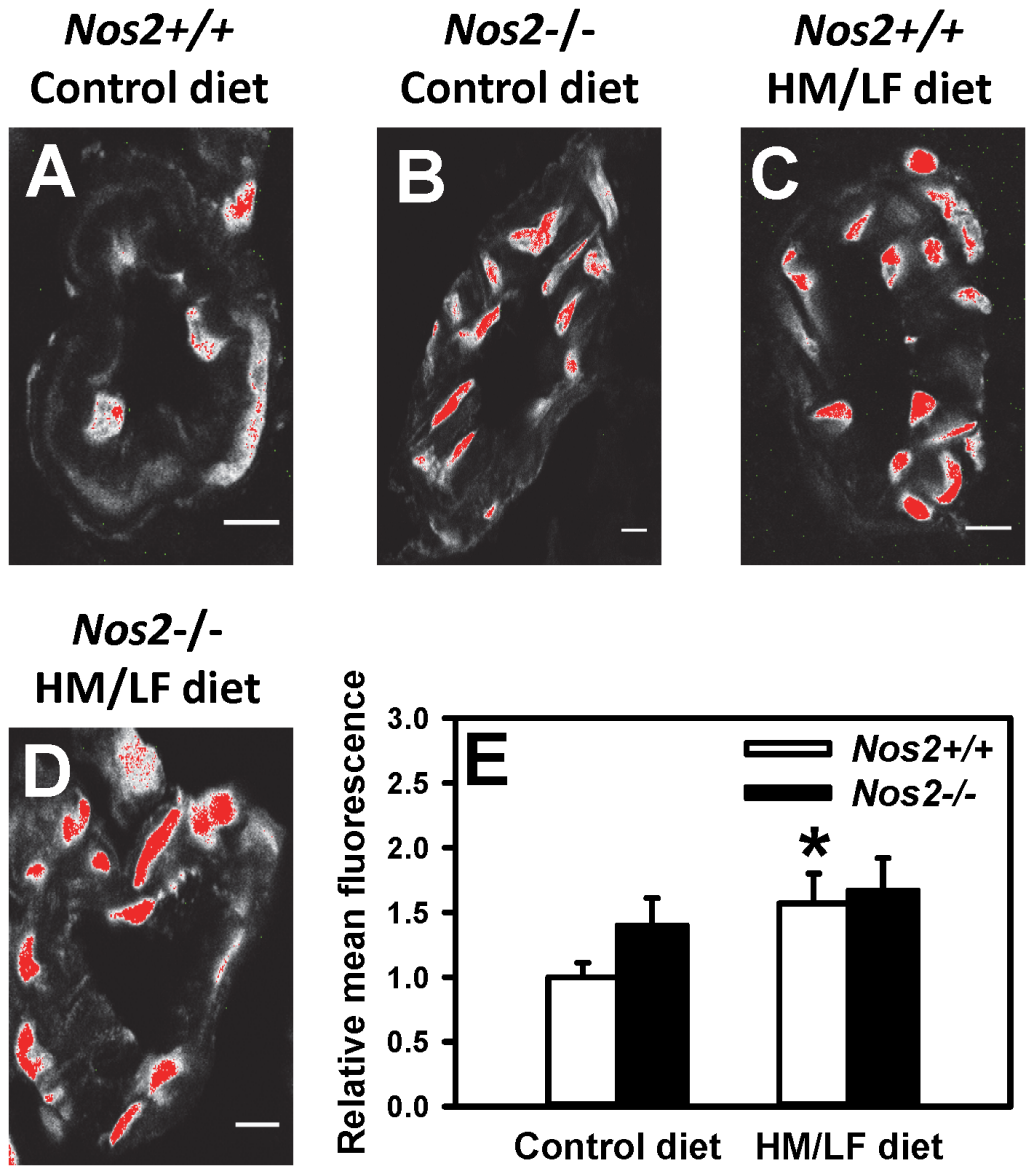
Laser-scanning confocal micrographs of cerebral arterioles stained with DHE. (A) *Nos2*+/+ mice fed the control diet. (B) *Nos2−/−* mice fed the control diet. (C) *Nos2+/+* mice fed the HM/LF diet. (D) *Nos2−/−* mice fed the HM/LF diet. (E) Quantification of DHE fluorescence using Image J. Images are representative of 5 separate experiments. Scale bar  = 10 µm.

Previous work has suggested a possible mechanistic role for vascular ROS in both the endothelial vasomotor dysfunction and prothrombotic state of diet-induced hyperhomocysteinemia [3]. Therefore, we utilized laser-scanning confocal microscopy to detect ROS in cerebral arterioles by DHE fluorescence. We found that DHE fluorescence was significantly (P<0.05) higher in *Nos2*+/+ mice fed the HM/LF diet (Figure 4C) compared with *Nos2*+/+ mice fed the control diet (Figure 4A). Deficiency of iNOS tended to increase DHE fluorescence in *Nos2*−/− mice fed the control diet (P = 0.07) (Figure 4B) and was increased further in *Nos2*−/− mice fed the HM/LF diet (Figure 4D). The quantitative data are summarized in Figure 4E. These findings suggest that endogenous *Nos2* protects from ROS generation rather than acting as a source of vascular ROS. Our findings also suggest that both iNOS deficiency and hyperhomocysteinemia independently contribute to increased vascular ROS.

### Hyperhomocysteinemia exacerbates myocardial I/R injury in iNOS-deficient mice

**Figure 5:**
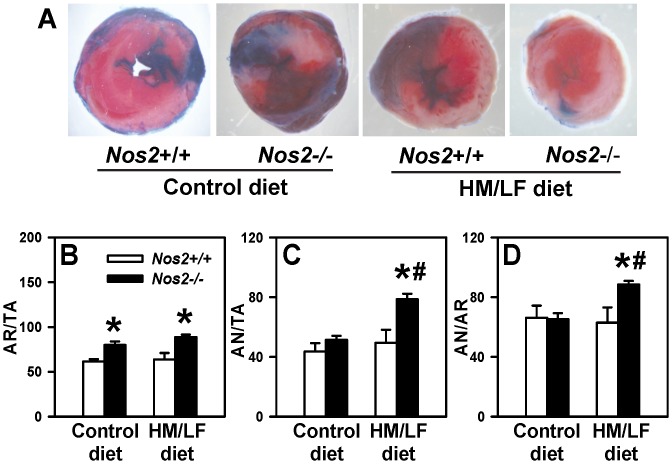
Regional myocardial ischemia-reperfusion injury. The left coronary artery was ligated for 30 minutes and then reperfused for 2 hours. Panel A illustrates representative 2,3,5-triphenyltetrazolium chloride (TTC) stained images for each of the four groups of mice. The percent of (B) area at risk over total area (AR/TA), (C) area of necrosis over total area (AN/TA), and (D) area of necrosis over area at risk (AN/AR) were calculated in *Nos2+/+* (open bars) or *Nos2−/−* mice (filled bars) by staining with Evans blue and TTC. Values are mean ±SE. 8–12 mice were studied in each group. *P<0.01 compared with *Nos2+/+* mice fed the same diet, #P<0.05 compared with mice of the same genotype fed the control diet by two-way ANOVA.

We next examined the effect of *Nos2* deficiency on regional myocardial I/R injury (Figure 5A). In *Nos2−/−* mice, the ischemic area at risk (AR) following I/R injury was significantly increased compared with *Nos2+/+* littermate mice, regardless of diet (P<0.01, Figure 5B). In *Nos2+/+* mice, the HM/LF diet did not alter infarct size, calculated either as area of necrosis per total area (AN/TA) (Figure 5C) or area of necrosis per area at risk (AN/AR) (Figure 5D). In *Nos2−/−* mice, however, the HM/LF diet caused a significant increase in infarct size (Figure 5C and 5D; P<0.05 vs. *Nos2+/+* mice fed the HM/LF diet; P<0.05 vs. *Nos2−/−* mice fed the control diet). These data suggest that endogenous iNOS protects against reperfusion injury in the setting of hyperhomocysteinemia.

**Figure 6:**
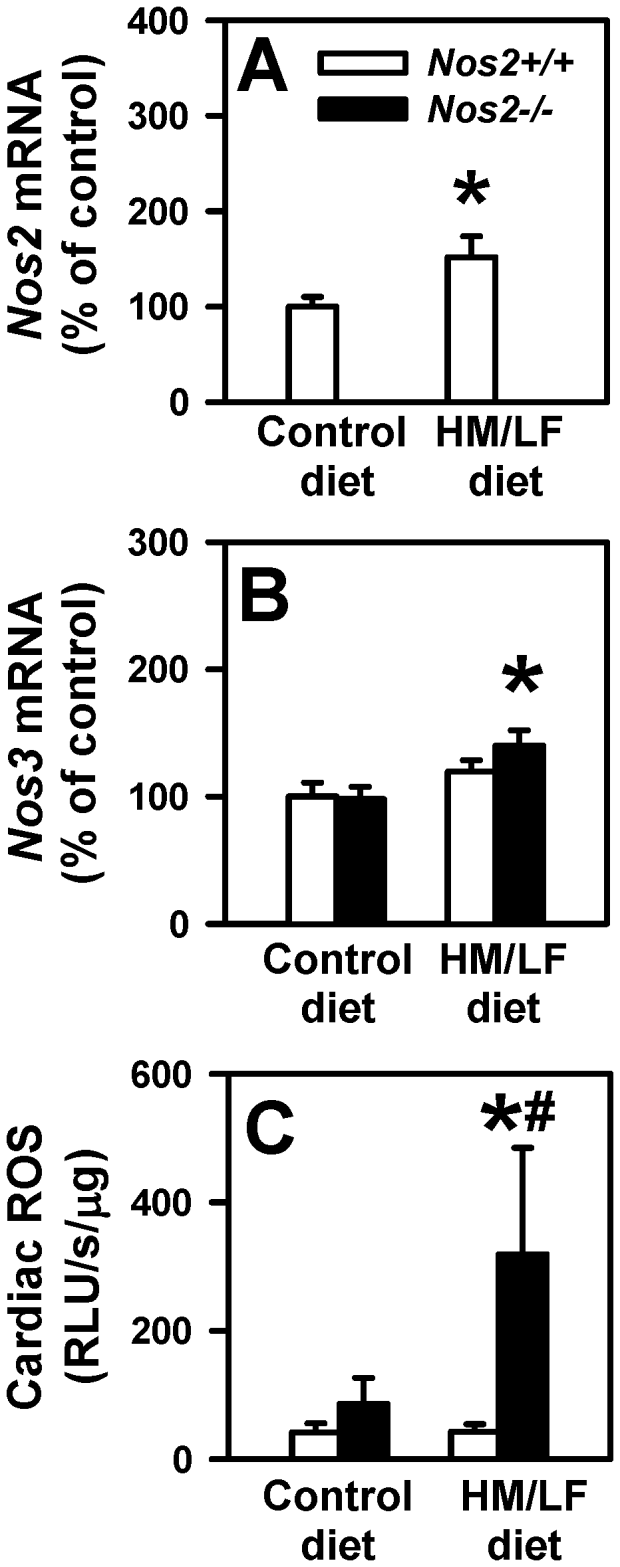
Cardiac levels of *Nos2* and *Nos3* mRNA and ROS. Levels of *Nos2* mRNA (A) and *Nos3* mRNA (B) were measured by real-time PCR in *Nos2+/+* (open bars) or *Nos2−/−* (filled bars) mice. Values were normalized to *18S* mRNA and are expressed as percent of the control values observed in *Nos2+/+* mice fed the control diet. (C) Tiron-quenchable and NADPH-enhanced lucigenin chemiluminescence was measured in cardiac homogenates. Values are mean ±SE. 6–9 mice were studied in each group. *P<0.05 compared with mice of the same genotype fed the control diet; #P<0.05 compared with *Nos2+/+* mice fed the same diet.

To further explore the relationship between NOS and ROS in the heart, we measured cardiac levels of *Nos1*, *Nos2* and *Nos3* mRNA by quantitative real-time PCR. The data demonstrate a 50% increase in *Nos2* mRNA levels in hearts from *Nos2*+/+ mice fed the HM/LF diet compared with the control diet (Figure 6A; P<0.05). This finding indicates that hyperhomocysteinemia induces the upregulation of iNOS expression in the heart, as has been previously reported in vascular tissue [22] and kidney [23]. As expected, no *Nos2* mRNA was detected in *Nos2*−/− mice (Figure 6A). There was no significant difference in *Nos3* mRNA expression between *Nos2*+/+ mice fed the control diet vs. the HM/LF diet (Figure 6B). We did detect a small but significant increase in *Nos3* mRNA levels in *Nos2*−/− mice fed the HM/LF diet (Figure 6B; P<0.05 vs. *Nos*2−/− mice fed the control diet). *Nos1* mRNA was not detected in cardiac tissue from any of the groups of mice (not shown). We next determined whether *Nos2* deficiency during hyperhomocysteinemia alters cardiac ROS production. A significant increase in cardiac ROS was observed in *Nos2*−/− mice fed the HM/LF diet (Figure 6C, P<0.05 compared with *Nos2−/−* mice fed the control diet; P<0.01 compared with *Nos2+/+* mice fed the HM/LF diet), consistent with the larger infarct size in this group (Figure 5B and 5C). These findings suggest that during hyperhomocysteinemia, endogenous iNOS protects cardiac tissue from oxidative stress and I/R injury.

## Discussion

In certain pathological conditions such as diabetes and atherosclerosis, iNOS has been proposed to be a mediator of increased oxidative stress and impaired vascular function. In diabetic mice, deficiency of iNOS protects against endothelial dysfunction [16], [17] and myocardial I/R injury [18], [19]. The absence of iNOS also decreases oxidative stress and protects against atherosclerosis in apolipoprotein E-deficient (ApoE−/−) mice [37]. Because hyperhomocysteinemia causes upregulation of vascular iNOS expression and increases vascular oxidative stress [22], [34], we hypothesized that genetic deficiency of iNOS also might protect against hyperhomocysteinemia-induced vascular dysfunction and thrombosis. To address this hypothesis, we examined the mechanistic role of iNOS in endothelial dysfunction, thrombosis and myocardial I/R injury in mice with or without diet-induced hyperhomocysteinemia. The major findings of our study are: 1) the absence of iNOS failed to protect mice against endothelial dysfunction or accelerated thrombosis produced by hyperhomocysteinemia; and 2) deficiency of iNOS exacerbated, rather than protected against myocardial ischemia-reperfusion injury and cardiac oxidative stress in hyperhomocysteinemic mice. These findings demonstrate that, unlike what has been described in models of diabetes or atherosclerosis, iNOS is not a mediator of the adverse vascular and endothelial effects of hyperhomocysteinemia.

Our data also suggest a baseline antithrombotic effect of endogenous iNOS in mice without hyperhomocysteinemia, demonstrated by a marked shortening of the time to thrombotic occlusion of the carotid artery in *Nos2−/−* mice fed the control diet (Figure 3). This observation is consistent with a prior study in which iNOS was found to provide protection against injury-induced thrombosis in female mice [38]. In our study, loss of endogenous iNOS also appeared to decrease dilatation responses to acetylcholine (Figure 2) and increase levels of ROS in cerebral arterioles (Figure 4) in the absence of hyperhomocysteinemia. Interestingly, a previous study found that *Nos2*−/− mice exhibited enhanced oxidative stress after traumatic brain injury [39]. These observations suggest that, rather than contributing to oxidative vascular injury, endogenous iNOS may protect against endothelial dysfunction and thrombosis by suppressing vascular oxidative stress.

Clinical evidence for increased troponin levels after coronary artery bypass in hyperhomocysteinemic patients suggests a relationship between elevated homocysteine and cardiac injury [40]. *Ex vivo* studies in isolated Langendorff-perfused hearts from mice with heterozygous deficiency of cystathionine β-synthase suggested that elevated homocysteine results in impaired relaxation and contractile function and increased apoptosis following I/R injury [4]. Unexpectedly, we did not observe a difference in infarct size in wild-type mice fed control vs. HM/LF diet. Potential reasons for the different findings between the *ex vivo* Langendorff results and our *in vivo* data include the different endpoints for myocardial injury examined, and that the *ex viv*o studies were conducted with a genetic model of hyperhomocysteinemia [4], whereas we used a diet-induced model of hyperhomocysteinemia.

Our findings in the myocardial I/R model are particularly interesting, since prior reports have suggested contrastingly that either deficiency or overexpression of iNOS can protect against I/R injury in mouse models [19], [41], [42]. We observed that the myocardial area at risk was increased with iNOS deficiency regardless of diet, suggesting that endogenous iNOS may limit the area of myocardial ischemia by promoting vasodilation of collateral blood vessels. We also found that cardiac iNOS mRNA was significantly upregulated by hyperhomocysteinemia in *Nos2+/+* mice (Figure 6A). Our data further suggest that the elevated levels of cardiac iNOS in hyperhomocysteinemic mice are protective against oxidative stress and I/R injury, because *Nos2−/−* mice had increased cardiac ROS production (Figure 6C) and increased infarct size (Figure 5) compared with *Nos2+/+* mice in the setting of hyperhomocysteinemia. This protective effect of iNOS is in accordance with previous studies in which gene transfer of iNOS protected against myocardial injury [42].

Our study has some limitations, including the inherent weaknesses of mouse models of human disease. The mouse model of diet-induced hyperhomocysteinemia, and the experimental models of vascular injury utilized herein may only approximate conditions that occur in human patients. Moreover, it is possible that some or all of the vascular effects observed in this study may be driven by metabolic consequences of the HM/LF diet that are independent of hyperhomocysteinemia. We note that the HM/LF diet produced only modest hyperhomocysteinemia in mice, with levels of plasma tHcy (10.3±0.9 µmol/L) somewhat lower than those seen in human patients with moderate hyperhomocysteinemia (10–20 µmol/L) and vascular diseases [1], [2]. It may be interesting in future studies to explore the vascular effects of additional dietary and genetic models of altered homocysteine, methionine and folate metabolism.

In summary, our data suggest a model in which, under normal or minimally pathological conditions (e.g. mild hyperhomocysteinemia, which is common in the general population), iNOS expressed at low levels in vascular cells is primarily protective against vascular oxidative stress and its complications. This vasoprotective effect of iNOS contrasts with its harmful effects in more severe pathological conditions (e.g. diabetes, atherosclerosis, or sepsis), in which upregulation of iNOS can mediate increased oxidative stress and related vascular pathology [16]–[19], [37], [43]. Our study design was comprehensive in that we examined the modulating effects of iNOS deficiency in both vascular and cardiac tissues in mice with diet-induced hyperhomocysteinemia. Our findings argue strongly against a role for iNOS as a mediator of the vascular, thrombotic, or cardiac complications of hyperhomocysteinemia in this mouse model.
